# Multiclassifier combinatorial proteomics of organelle shadows at the example of mitochondria in chromatin data

**DOI:** 10.1002/pmic.201500267

**Published:** 2016-01-25

**Authors:** Georg Kustatscher, Piotr Grabowski, Juri Rappsilber

**Affiliations:** ^1^ Wellcome Trust Centre for Cell BiologyUniversity of EdinburghUK; ^2^ Department of BioanalyticsInstitute of BiotechnologyTechnische Universität BerlinBerlinGermany

**Keywords:** Chromatin, Machine learning, Mitochondria, Organelle, Systems biology

## Abstract

Subcellular localization is an important aspect of protein function, but the protein composition of many intracellular compartments is poorly characterized. For example, many nuclear bodies are challenging to isolate biochemically and thus remain inaccessible to proteomics. Here, we explore covariation in proteomics data as an alternative route to subcellular proteomes. Rather than targeting a structure of interest biochemically, we target it by machine learning. This becomes possible by taking data obtained for one organelle and searching it for traces of another organelle. As an extreme example and proof‐of‐concept we predict mitochondrial proteins based on their covariation in published interphase chromatin data. We detect about ⅓ of the known mitochondrial proteins in our chromatin data, presumably most as contaminants. However, these proteins are not present at random. We show covariation of mitochondrial proteins in chromatin proteomics data. We then exploit this covariation by multiclassifier combinatorial proteomics to define a list of mitochondrial proteins. This list agrees well with different databases on mitochondrial composition. This benchmark test raises the possibility that, in principle, covariation proteomics may also be applicable to structures for which no biochemical isolation procedures are available.

**Abbreviations**
ChEP: chromatin enrichment for proteomics
MCCP: multiclassifier combinatorial proteomics

## 1 Introduction

Eukaryotic cells contain organelles and other specialized compartments, whose protein composition can be analyzed by proteomics to provide important clues regarding their biological function 1, 2. Organelle proteomics approaches traditionally depend on the biochemical isolation of the analyzed structure, which can be relatively straightforward for membrane‐enclosed organelles such as mitochondria 3. However, the majority of spatial compartments cannot be adequately enriched for conclusive analysis, as their isolates may be contaminated with too many functionally unrelated proteins that copurify. Alternative approaches have therefore been developed to infer the composition of organelles that cannot be purified to homogeneity. For example, subtractive 4 and quantitative 5 proteomics approaches have been employed to distinguish between genuine components and contaminants in biochemical isolates of nuclear envelopes and lipid rafts, respectively. Partial enrichment combined with quantitative proteomic analysis was used to broadly categorize the cell into cytoplasm, nucleus, and nucleolus 6. Protein correlation profiling was developed to study the composition of the centrosome 7 and later provided a mammalian organelle map 8. Using a related method, localization of organelle proteins by isotope tagging, proteins were assigned to the various compartments of the endomembrane system, which cannot be efficiently distinguished biochemically 9.

**Significance of the study**

This study introduces a new concept for organelle proteomics. Until now, specific biochemical enrichment was paramount to study biological structures by proteomics. However, many compartments in the cell simply cannot be isolated or even partially separated from the rest of the cell. Examples for this include chromatin, which is highly charged and invariably “absorbs” functionally unrelated proteins, and nuclear bodies that are not surrounded by a membrane and most likely disintegrate upon cell lysis. We present here a method that may overcome such challenges in the future. The basic idea is that machine‐learning can identify organelle‐specific patterns across many comparative proteomics studies, even if the organelle was just present as contamination in the original experiment. As a proof‐of‐principle we identified mitochondrial proteins from chromatin proteomics data. While we do not have enough data at the moment to define the entire mitochondrial proteome in this way, our experiment shows that enriching an organelle through biochemical fractionation is no longer a strict requirement to analyze its composition. We envisage that this method may be useful to study a multitude of nonpurifiable biological structures in the future.

When analyzing mitotic chromosomes we also encountered an abundant presence of background proteins 10. Importantly, mitotic chromosomes are large and highly charged, attracting many functionally unrelated proteins, and thus are physically contaminated themselves. This made it difficult to identify contaminants using the existing fractionation‐based procedures. We therefore proposed a machine learning approach, multiclassifier combinatorial proteomics (MCCP), as a solution. Taking the outcome of multiple proteomic analyses of mitotic chromosomes that were done under biochemically or genetically distinct conditions, and integrating those by Random Forest analysis provided a ranked list of protein components of mitotic chromosomes. Interphase chromatin is another example of a specialized functional compartment whose biochemical isolates remain highly impure 11. Working with partially purified material, we used MCCP to infer the protein composition of interphase chromatin from biological covariation. For this we analyzed chromatin‐enriched samples from a wide variety of biological conditions and showed that proteins with well‐known chromatin functions tend to respond in a similar way to various perturbations, such as drug treatments. We subsequently used a machine learning algorithm to capture the covariation pattern corresponding to chromatin factors. The resulting model allowed us to predict hundreds of new potential interphase chromatin proteins simply based on their covariation with already known chromatin proteins.

Some compartments may be inherently unstable in vitro. For example, it has been proposed that many intracellular bodies represent liquid droplets that form by phase transition from the surrounding cyto‐ or nucleoplasm 12. Such compartments may be very difficult or even impossible to purify biochemically, and presumably would start to disintegrate after cell lysis. Therefore, new approaches may be required to determine their protein composition. Possibly, also here a solution could come from machine learning.

One conclusion from our analysis of interphase chromatin was that covariation with reference proteins was more accurate than biochemical enrichment in identifying chromatin components. This raised the intriguing possibility that biochemical enrichment may not be an essential element of determining the composition of cellular structures by proteomics. To push this hypothesis to its extreme we wondered if an entirely untargeted organelle could be defined through its changing coappearance in the analysis of chromatin. This would offer a way to study the composition of nonpurifiable compartments, especially that of many elusive nuclear bodies that may stick to chromatin when it is isolated but that cannot be isolated on their own.

To test the hypothesis that covariation in proteomic datasets can be the central element of studying the composition of cellular structures by proteomics, we attempted to define the composition of mitochondria on the basis of our chromatin proteomics dataset. Our intention was not to present an alternative or even superior way of analyzing mitochondria but simply to use mitochondria as a test system for other organelles or structures that challenge current analysis approaches. Mitochondria are large, well defined, and not functionally linked to chromatin in any obvious way, but are frequently part of the background of our chromatin enrichment procedure. We defined a high‐quality reference set of mitochondrial proteins and used this to train a machine‐learning algorithm to spot other mitochondrial proteins in our chromatin dataset. The results agreed well with the current consensus of which proteins are in mitochondria. We could not expect to obtain a comprehensive mitochondrial protein inventory, because only ⅓ of the known mitochondrial proteins were detected in our chromatin samples. However, this proof‐of‐principle experiment demonstrates the possibility that targeted biochemical enrichment may be optional and not essential for defining organelles. Subcellular localization may be predicted through covariation, thus allowing targeting a structure during data analysis rather than experimentally.

## 2 Materials and methods

### 2.1 Chromatin proteomics data

Proteomic analyses of interphase chromatin were described previously 11. For this project we only considered 45 SILAC ratios comparing chromatin under different biological conditions. Only those 4565 proteins with values in at least ten out of all 45 chromatin proteomics experiments were considered (Supporting Information Table 1). In brief, these experiments consisted of human cell lines grown in SILAC medium and subjected to various perturbations, such as treatment with drugs, growth factors, or irradiation. They also include SILAC‐based comparisons of different cell types and cell‐cycle phases. In order to preferentially detect chromatin‐bound proteins, all samples were subjected to the chromatin enrichment for proteomics (ChEP) procedure 13. Tryptic digests were analyzed by LC‐MS/MS on an LTQ‐Orbitrap or LTQ‐Orbitrap Velos (Thermo Fisher Scientific). These samples are described as “biological classifier” experiments in Table 1 of Kustatscher et al. 11 in more detail. Raw data have been deposited in the PRIDE 14 repository (www.ebi.ac.uk/pride) as part of the dataset PXD000493 (for this study we only used a subset of these data, namely experiments 3–7 and 18–35).

### 2.2 High‐confidence mitochondrial reference protein set

We compiled a set of well‐studied, high‐confidence mitochondrial reference proteins. As a starting point, we downloaded all 1065 human proteins that mapped to “mitochondrion” in Uniprot's 15 subcellular localization database (www.uniprot.org/locations) and that were part of Swiss‐Prot. We kept only proteins with an annotation score of at least four out of five. To remove proteins with ambiguous localization we filtered out proteins whose localization annotation matched the following keywords: nucleus, reticulum, Golgi, secreted, cytosol, peroxisome, and cell projection. This shortlisted 653 proteins, for which we manually evaluated Uniprot and GO 16 annotations and, where necessary, searched the available literature to extract a final list of 486 bona fide mitochondrial proteins with no reported functions elsewhere in the cell. Of these 486 mitochondrial reference proteins, 172 (35%) were detected in the chromatin proteomics dataset (Supporting Information Table 2).

### 2.3 Random Forest prediction of mitochondrial proteins

For supervised machine learning we used the Weka 3.7 17 implementation of the Random Forest algorithm 18, executed through an in‐house workflow built on the KNIME data analytics platform 19. This implementation of Random Forest does not impute missing values. The Random Forest was constructed using 500 trees, six random features at each split and an unlimited maximum tree depth. The high‐confidence mitochondrial reference protein set was used as positive training data. Negative training data were randomly selected from all nonmitochondrial proteins in our chromatin proteomics dataset (for this purpose, nonmitochondrial was defined as having no such annotation in GO or Uniprot). To avoid using unbalanced training data, only 172 negative training instances were selected, i.e. the same number as positive training instances. However, rather than constructing just one Random Forest, the workflow was executed ten times with different randomly drawn negative training data. The average Random Forest scores and their standard deviation were collected. Prediction accuracy was assessed in two different ways. The out‐of‐bag error, an unbiased estimate of the test set error inbuilt to the algorithm, was collected. In addition, the training dataset was cross‐validated 100‐fold, and the cross‐validated data were used to judge performance based on the area under a ROC curve. Random Forest scores, including the cross‐validated scores for the mitochondrial training dataset, are reported in the Supporting Information Table 2.

### 2.4 Comparison with other mitochondrial datasets

We compared our mitochondrial predictions to five different sources of mitochondrial annotation. The human version of MitoCarta 20 was downloaded on May 1, 2015 from www.broadinstitute.org/pubs/MitoCarta. GO annotations 16 were downloaded from QuickGO 21 using the identifier “mitochondrion” (GO:0005739), restricted to the qualifiers “contributes to,” “colocalizes with” and “none”. Only annotations with evidence level “manual experimental” were considered. The third external mitochondrial protein set consisted of proteins annotated as mitochondrial in Uniprot and was downloaded as described for the high‐confidence mitochondrial reference protein set, without filtering against multiple localizations. An immunofluorescence‐based list of proteins with mitochondrial localization was retrieved from the Human Protein Atlas 22, omitting proteins with “uncertain” reliability status. The fifth reference set consisted of mitochondrial matrix proteins identified via spatially restricted enzymatic tagging and MS 23.

### 2.5 Further data processing and visualization

Data were processed using R version 3.2 24. ID conversions, where necessary, were done using Bioconductor biomaRt package 25. ROC curves were generated and visualized using ROCR R package 26. Data analysis plots were prepared using ggplot2 R package 27.

## 3 Results and discussion

Covariation proved to be a successful concept in defining core proteins of mitotic and interphase chromatin when starting from multiple but impure proteomic lists of these structures 10, 11. To test if this approach could be expanded to structures that have not been the target of experimental data collection we attempted here to define mitochondria through their coappearance in chromatin analyses. We chose mitochondria for this proof‐of‐principle experiment, because this organelle has been well‐defined through other studies and thus allows us to evaluate the success of our approach. Mitochondria are membrane‐enclosed and thus presumably clearly defined, and their composition has been studied for decades with many different experimental approaches, including proteomics. This makes them a good reference point to assess the performance of novel organelle proteomics approaches. Moreover, mitochondria contain more than thousand proteins 20, several hundred of which are detected in our chromatin samples, providing a reasonably sized test set for our setup. It should be noted that some mitochondrial proteins, such as prohibitins, have genuine additional functions as nuclear transcription factors and so would be expected to be found in chromatin 28. However, the majority of mitochondrial proteins in our assay likely become associated with chromatin in an artificial way at some point during chromatin enrichment, i.e. they are likely contaminants in our chromatin analysis.

### 3.1 Biological perturbations affect the abundance of mitochondrial proteins in chromatin samples

**Figure 1:**
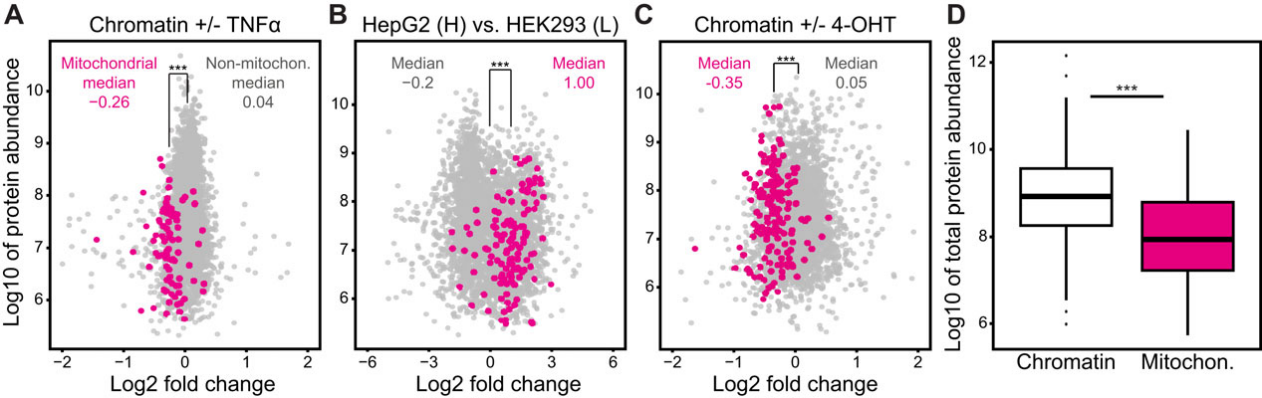
Mitochondrial proteins in interphase chromatin samples. (A–C) Mitochondrial proteins (magenta) are present in chromatin proteomics data, and are up‐ or downregulated in response to biological perturbations. For example, they are downregulated after treating HeLa cells for 10 min with TNFα compared to untreated controls (A). They are upregulated in chromatin samples purified from HepG2 as opposed to HEK293 cells (B). Mitochondria are also depleted from chromatin samples after treating estradiol‐treated MCF7 cells with 4‐hydroxytamoxifen (4‐OHT) (C). The fold change is the SILAC ratio and protein abundance is the sum of measured peptide intensities. The significance of mitochondrial fold‐changes was evaluated by *t*‐test and yielded *p*‐values < 0.001 in all three cases, as illustrated by the triple asterisks. (D) Boxplot of protein abundances showing that chromatin proteins are nearly an order of magnitude more abundant than mitochondrial (Mitochon.) proteins, supporting the contaminant status of the latter. The sum of protein intensities measured across all experiments is plotted. The intensity difference is highly significant according to a *t*‐test (*p*‐value *=* 5.4 × 10^–32^).

We observed that the presence of mitochondria in chromatin samples tends to change—very gently—in response to biological perturbations (Fig. 1). This is initially counter‐intuitive as one would expect from a contaminating protein that its presence would be largely unaffected by biological changes in chromatin. Surprisingly, mitochondrial proteins become mildly but significantly depleted (*p* = 1.13 × 10^–10^) in chromatin samples after treating cells with TNFɑ (Fig. 1A), they are more abundant (*p* = 7.26 × 10^–22^) in chromatin samples from HepG2 than HEK293 cells (Fig. 1B) and they are depleted (*p* = 7.95 × 10^–30^) from chromatin following 4‐hydroxytamoxifen treatment (Fig. 1C). Indeed, in most comparative chromatin proteomics experiments, we find that mitochondria are slightly enriched or depleted in one condition compared to the other (Supporting Information Table 3). These changes are likely due to the primary or secondary effects of a perturbation on the cell, although we can only speculate about the precise mechanisms involved. For example, alterations in chromatin structure may affect the association of background proteins, leading to increased or decreased copurification of mitochondria with chromatin under different conditions. In addition, the number of mitochondria per cell may also be altered in some experiments, e.g. when comparing different cell types. While it is difficult to pinpoint the exact reasons for mitochondrial abundance variation in chromatin samples, we set out to test whether these changes can be exploited to study mitochondrial proteins.

### 3.2 Mitochondria are not major contaminants in chromatin samples

To ensure that mitochondria are a valid initial test system for our method, we first confirmed that mitochondria are not preferentially coenriched with chromatin. First, we noted that mitochondrial proteins are nearly an order of magnitude less abundant than chromatin proteins in these samples (Fig. 1D). To further confirm their status as contaminants, we turned to our chromatin proteome study, in which we assigned probabilities for genuine chromatin‐based functions to human proteins. As expected, the vast majority of mitochondrial proteins (94%) are not predicted to have a functional association with chromatin (Supporting Information Fig. 1A). Finally, we tested how mitochondrial abundance in chromatin samples compares to that of various other organelles and common contaminants, such as ribosomes, the cytoskeleton and the Golgi apparatus. In fact, mitochondria are the least abundant of the tested chromatin contaminants (Supporting Information Fig. 1B).

### 3.3 Covariation in chromatin samples can predict mitochondrial proteins

We previously observed coordinated bulk behavior for chromatin proteins versus background proteins across various biological situations 11. This covariation of chromatin factors allowed us to construct a comprehensive inventory of interphase chromatin. We defined a reference set of known chromatin factors and then used a Random Forest machine learning algorithm to identify proteins with similar behavior across various chromatin proteomics experiments. Now, we tested whether this approach could also capture a mitochondria‐specific pattern across the same set of chromatin proteomics experiments.

We first assembled a high‐confidence set of mitochondrial proteins. We started from a list of proteins annotated as mitochondrial in Uniprot and removed all entries with potentially ambiguous subcellular localization, such as mitochondrial proteins with additional reported functions in the endoplasmic reticulum or elsewhere in the cell. For this we considered information from Uniprot, GO, and the primary literature. We further removed proteins which were generally not well characterized, and could therefore not be considered bona fide mitochondrial proteins. Of the remaining 486 proteins we observed 172 (35%) in our data. We also sought to define a high‐confidence set of nonmitochondrial proteins without introducing a bias. Such a bias could result from simply selecting nuclear proteins, for example. We solved this by drawing nonmitochondrial proteins randomly from all proteins in our dataset, except from proteins that had mitochondrial annotations in either Uniprot or GO.

We then conducted a supervised machine learning experiment based on the Random Forest algorithm 18 to distinguish mitochondrial from nonmitochondrial proteins using a publically available chromatin proteomics dataset (ProteomeXchange PXD000493) 11. The dataset was obtained by analyzing chromatin‐enriched samples from human cell lines grown in SILAC medium and subjected to various perturbations, such as treatment with drugs, growth factors, or irradiation. They also include SILAC‐based comparisons of different cell types and cell‐cycle phases. In order to preferentially detect chromatin‐bound proteins, all samples were subjected to the ChEP procedure 13. The chromatin dataset comprised 23 double‐ and triple‐SILAC experiments with 45 SILAC ratios in total (Supporting Information Table 1). The Random Forest was trained using the reference sets of mitochondrial and nonmitochondrial proteins. For the nonmitochondrial training set to be representative of most nonmitochondrial cellular compartments we would have had to use significantly more than 172 proteins, as we used for the mitochondrial training set. However, using unbalanced training data skews the resulting scores. We therefore opted to train ten Random Forests, each time with the same 172 mitochondrial proteins but a different set of 172 randomly chosen nonmitochondrial training proteins. We collected the average Random Forest scores for each protein. This approach has the advantage of using a balanced training set and still sample a large fraction of all nonmitochondrial proteins to minimize prediction bias. In addition, the standard deviation of the score across the ten different Random Forest models reveals the impact of the choice of nonmitochondrial training proteins. The resulting set of Random Forests could distinguish between the known mitochondrial and nonmitochondrial training proteins very well, as indicated by the mean out‐of‐bag error of 0.1 ± 0.008. This shows that we can identify mitochondrial proteins only based on their SILAC ratios across many chromatin proteomics experiments.

**Figure 2:**
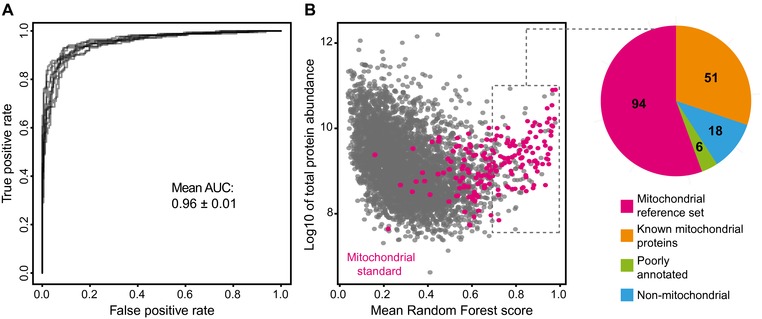
A Random Forest model can predict mitochondrial proteins based on their covariation in chromatin proteomics data. (A) High accuracy of mitochondrial prediction is shown by ROC curves derived from the 100‐fold cross‐validated mitochondrial and nonmitochondrial reference set. The ten curves correspond to ten Random Forests generated with different negative training data, highlighting the robustness of the Random Forest model. AUC: area under the curve. (B) Random Forest scores for the 4565 proteins (gray) in our analysis. High‐confidence mitochondrial reference proteins (magenta) are heavily enriched toward higher scores. The pie‐chart shows the manual annotation of proteins within the dashed rectangle, corresponding to a score cut‐off of 0.69. Most proteins are either part of our high‐confidence mitochondrial reference set or other known mitochondrial proteins. Six proteins were poorly annotated. 18 proteins were classified as nonmitochondrial, i.e. they are well‐annotated but no evidence for mitochondrial function exists. This group was used to estimate that we have about 10% false positives at this score cut‐off.

We next performed 100‐fold cross‐validation to determine reliable prediction scores for our high‐confidence mitochondrial proteins. This means we constructed 100 Random Forests and in each left out a different 1% of the reference data, using the model generated with the remaining 99% to obtain unbiased prediction scores for these proteins. This allowed us to use a ROC curve to estimate the model's performance, in addition to the inbuilt out‐of‐bag error estimate of the Random Forest algorithm. The mean area under the ROC curve we obtained was 0.96 (Fig. 2A). This confirms the high accuracy of our prediction already indicated by the low out‐of‐bag error.

In addition to our reference mitochondrial proteins, many other proteins with known mitochondrial functions received high Random Forest scores (Fig. 2B). To evaluate our prediction accuracy in a systematic way, we searched for false positive predictions among our top hits. For this we manually assessed the available literature and labeled proteins as false positives if they were well‐characterized but lacked evidence for mitochondrial localization. At a Random Forest score cut‐off of 0.69 we had 169 proteins of which 18 were clearly not mitochondrial (∼10% false positives). Of the remaining 151 proteins (Fig. 2B), 94 are part of our high‐confidence mitochondrial protein set and an additional 51 proteins are known to be mitochondrial. Six proteins were poorly or ambiguously annotated. For example, the prolyl hydroxylase LEPRE1 has one isoform that is thought to be secreted 29 and another one that may reside in mitochondria 30, but our data do not allow us to distinguish between the two. The other five proteins are candidates for novel mitochondrial proteins, warranting further study into their biological function.

### 3.4 Mitochondria predictions agree well with established mitochondrial protein inventories

**Figure 3:**
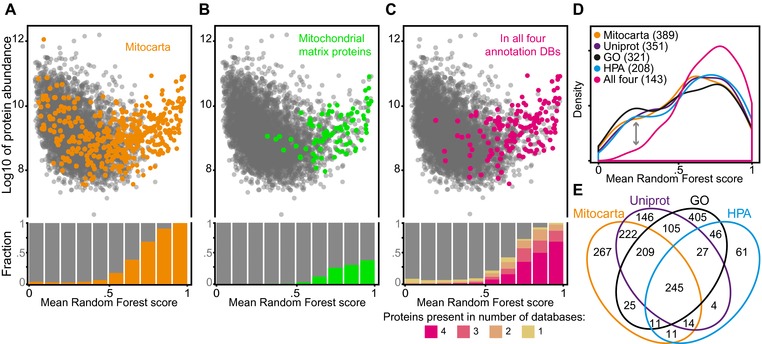
Covariation‐based prediction evaluated by comparison to existing mitochondrial protein inventories. (A) Mitochondrial prediction for all 4565 proteins is shown as their Random Forest machine learning score. Proteins that are present in MitoCarta 20 are highlighted in orange. There is a strong enrichment of MitoCarta proteins toward high Random Forest scores (see bar chart). (B) Same plot but highlighting proteins in green that were specifically assigned to the mitochondrial matrix by Rhee et al. 23. Note that very few of these annotations receive low Random Forest scores. Many high scoring proteins are mitochondrial but not in Rhee et al.’s 23 matrix proteome. (C) Proteins in magenta are annotated as mitochondrial in four different databases: MitoCarta, Uniprot, GO, and the Human Protein Atlas (HPA). These overlapping, high‐confidence annotations include fewer low‐scoring predictions than proteins found in only 1, 2, or 3 of these databases (see bar chart). (D) Distribution of mean Random Forest scores for proteins annotated as mitochondrial in either of the four indicated databases. Individually, all databases show a bimodal distribution. Restricting the analysis to the overlapping annotations shows a marked reduction in low‐scoring annotations (see arrow). This indicates that such proteins are not just missed in our prediction due to lack of sensitivity, but are also not supported by other databases. (E) Venn diagram depicting the overlap of mitochondrial annotations in the four databases, including proteins not detected in our dataset.

To determine the specificity and sensitivity of our approach in more detail, we compared its predictions to existing mitochondrial annotation data (Fig. 3). The most comprehensive inventory of mitochondrial proteins yet, MitoCarta, combined proteomic analysis of isolated mitochondria with GFP tagging and microscopy, and included additional genome‐scale datasets such as the occurrence of mitochondrial targeting sequences 20. The vast majority of proteins that receive high mitochondrial scores in our study are indeed found in MitoCarta, confirming the high specificity of our predictions (Fig. 3A). There is also strong enrichment of MitoCarta proteins toward high Random Forest scores. However, a number of MitoCarta proteins do not score high in our approach, raising the possibility that “prediction by covariation” may lack sensitivity. Alternatively, low‐scoring proteins in our model may have been falsely assigned to mitochondria by classical proteomic approaches, for example due to an artificial copurification with mitochondria‐enriched biochemical fractions. To test this possibility, we followed three separate lines of evidence.

First, we compared MitoCarta's confidence measure, the Maestro score, to our Random Forest score. MitoCarta entries which scored low in our analysis also tend to have been assigned to MitoCarta with lower confidence (Supporting Information Fig. 2). Next, we compared our predictions to a second, independent proteomic dataset that targeted proteins of the mitochondrial matrix rather than the entire mitochondrion 23. In this approach, a genetically modified peroxidase enzyme is fused to a localization signal that specifically targets it to the mitochondrial matrix, where it biotinylates proteins in close physical proximity. This method results in very high specificity, because the inner mitochondrial membrane acts as a barrier confining the biotin label to matrix proteins. Interestingly, when compared to our Random Forest predictions, there are far fewer low‐scoring proteins among mitochondrial factors identified in this way (Fig. 3B). This is also exemplified by a shift of median Random Forest score from 0.60 for MitoCarta proteins to 0.76 for mitochondrial proteins listed by Rhee et al. 23.

For a third specificity test, we compiled a consensus list of mitochondrial proteins by integrating four subcellular localization databases: MitoCarta, Uniprot, GO, and the Human Protein Atlas 15, 16, 20, 22. There was complete agreement among the four databases on 245 proteins. One hundred forty‐three of these we observed in our study. Similar to the matrix annotations from Rhee et al. 23, we find that the vast majority of these 143 consensus proteins rank very high in our predictions (median Random Forest score 0.74) (Fig. 3C). Interestingly, any individual database contains a number of mitochondrial annotations that receive low scores in our assay (Fig. 3D). Increasing the number of databases that must agree on a protein to be mitochondrial decreases the number of low scoring annotations and improves the median Random Forest score (any database: 0.27, any two databases 0.46, any three databases: 0.63, all four databases: 0.74).

These three points suggest that our Random Forest analysis succeeds in recognizing bona fide components of mitochondria. Scoring low in our analysis indicates that a protein is less likely to be a genuine component of mitochondria. A conclusive evaluation of false negatives in our analysis is complicated by an absence of large consensus on mitochondrial proteins. A total of 1798 proteins are labeled “mitochondrial” by at least one database while the four databases agree on only 245 (Fig. 3E). However, two reasons could account for genuine mitochondrial proteins scoring low in our assay. First, the accuracy of the Random Forest classification depends on the number of experiments available to it, so increasing the number of input experiments will increase performance further. Also, we cannot expect to identify “conditional” mitochondrial factors, i.e. proteins that only localize to mitochondria under certain biological conditions. This is because such proteins may have a predominant function elsewhere in the cell and therefore not covary with mitochondrial reference proteins.

Due to the low coverage of mitochondrial proteins in the chromatin dataset, we are unable to make predictions on the majority of the estimated 1129 mitochondrial proteins 20. For example, we detected 389 (38%) of the 1013 proteins in human MitoCarta. Therefore, we cannot carry out a comprehensive analysis of the entire organelle and cannot match existing resources like MitoCarta in terms of completeness. Most published proteomics data now become available through repositories such as PRIDE, so we expect that in the future it will be possible to mine much larger datasets for mitochondrial proteins in this way. While we only show here the example of mitochondria in chromatin samples, we would expect that, in principle, any comparative proteomics experiment could be used as input dataset, as long as some components of the target structure have been detected and accurately quantified in it. It should be noted that with this method no individual experiment needs to strongly separate the target structure from the rest of the cell, but separation is achieved by integrating many small, apparently insignificant differences into one machine learning score.

### 3.5 Feature count influences prediction accuracy

One important parameter affecting the accuracy of mitochondrial predictions by machine learning is the “feature count,” i.e. the number of different experiments in which a protein was quantified. The more feature counts (SILAC ratios) are available to establish the “covariation pattern” of a protein, the better a protein can be assigned to a certain functional group. For example, some of the 143 mitochondrial consensus proteins, on which all annotation databases agree, remain below our mitochondrial prediction cut‐off. These mitochondrial proteins have been quantified in a median of 16 ± 7 SILAC experiments. By contrast, the consensus proteins that score above cut‐off and are thus successfully predicted to be mitochondrial, have a median of 22 ± 9 features, and this difference is statistically significant (*p*‐value < 0.001).

Our mitochondrial protein predictions are based on SILAC data, i.e. relative rather than absolute protein abundances. This implies that protein abundance itself should not have a direct impact on prediction accuracy, but there is arguably an indirect effect of protein abundance on performance. For example, abundant proteins will generally be observed more often, resulting in higher feature counts. SILAC quantitation itself also tends to be more accurate for abundant proteins.

### 3.6 Implications for the design of SILAC studies

The observation that background in SILAC experiments changes systematically has implications for the design of comparative proteomics studies. For example, studies that test the effect of a drug on chromatin proteins would typically compare chromatin fractions from treated samples with a mock control and may conclude that all measured changes relate to the drug's effect on chromatin composition. However, our observations suggest that care should be taken when drawing such conclusions. Changes among the purification background, either through direct or indirect effects of the perturbation, are in fact widespread. This is illustrated by the fact that mitochondria can be up‐ and downregulated significantly in chromatin samples between experiment and control (Fig. 1A–C). We made a similar observation in a screen for novel DNA replication factors, where we isolated nascent chromatin using an unrelated biochemical procedure 31. Upon comparing nascent and mature chromatin we observed many differences that were clearly unrelated to chromatin replication. These rather reflected alterations in chromatin association of background proteins. To obtain high‐confidence DNA replication factors we filtered the data using interphase chromatin probabilities 11.

## 4 Concluding remarks

We provide proof‐of‐principle data to show that background in comparative proteomics experiments is not static or random, but exhibits fluctuations that are possibly biologically meaningful and can, in fact, be exploited. Background proteins with similar functions, such as mitochondrial factors, are coordinately up‐ or downregulated in chromatin analyses. We demonstrate that this makes it possible to predict components of mitochondria based solely on their behavior in chromatin samples, by quantifying their presence across a diverse range of conditions and using machine learning to compare it to reference proteins of known function. In principle, we would expect our approach to work for any organelle or compartment that has been detected in quantitative proteomics data although this remains to be demonstrated. With specific significance to nuclei, a large number of nuclear bodies have been difficult to purify on their own and may well be seen as “shadows” in our chromatin data. Future work will have to show if shadow proteomics provides a path to mapping these and other elusive structures in cells.

*The authors have declared no conflict of interest*.

## Supporting information

As a service to our authors and readers, this journal provides supporting information supplied by the authors. Such materials are peer reviewed and may be re‐organized for online delivery, but are not copy‐edited or typeset. Technical support issues arising from supporting information (other than missing files) should be addressed to the authors.

Supplementary Material
