# Supplementary figures and images for: Multiclassifier combinatorial proteomics of organelle shadows at the example of mitochondria in chromatin data
Source: Proteomics. 2016 Jan 25;16(3):393–401. doi: 10.1002/pmic.201500267 (PMC4862026; doi:10.1002/pmic.201500267)

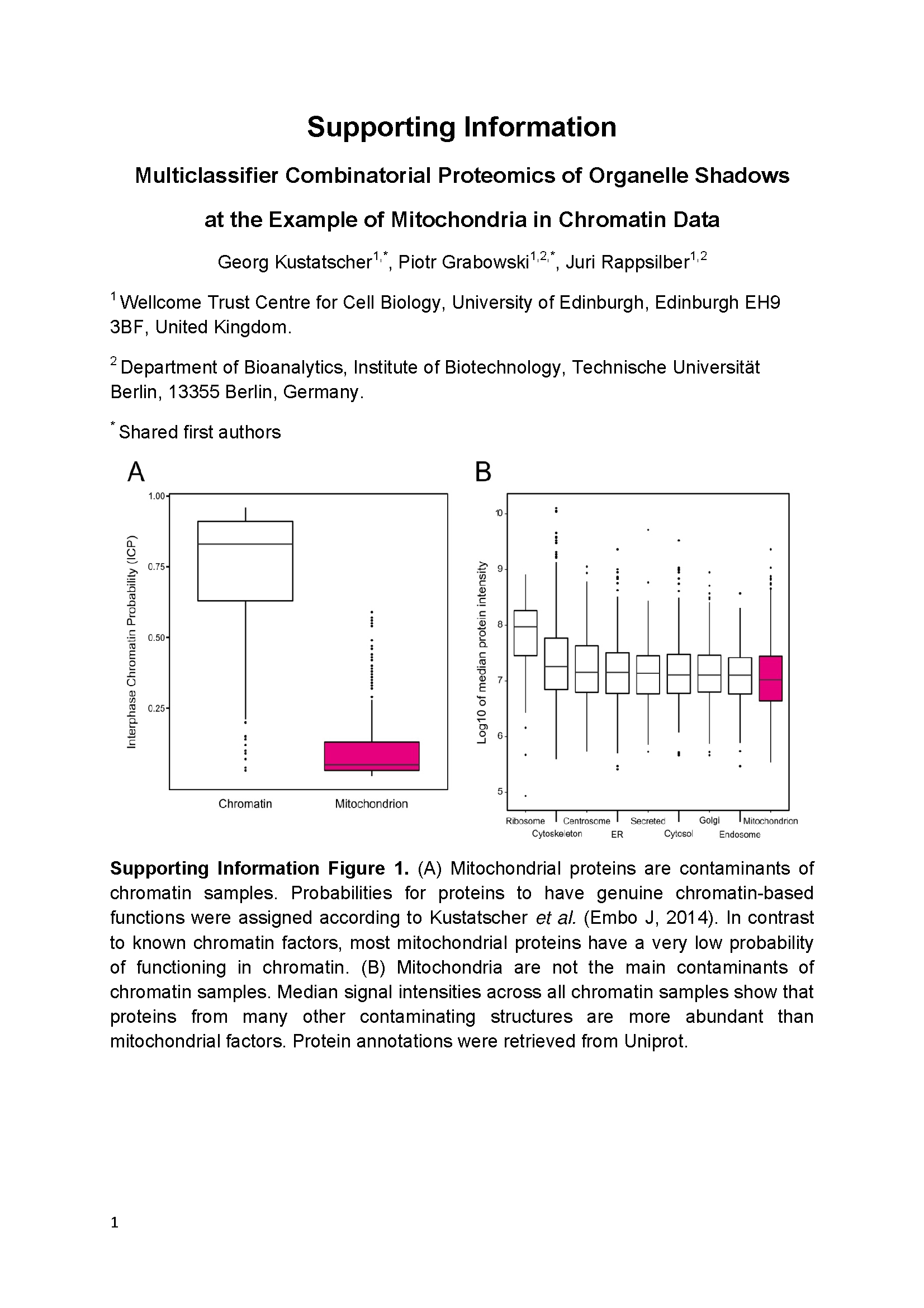

Supplement: Supplementary file 1 — Supplementary Material [file PMIC-16-393-s001.zip › pmic12175-sup-0001-Supporting_Informati.jpg]

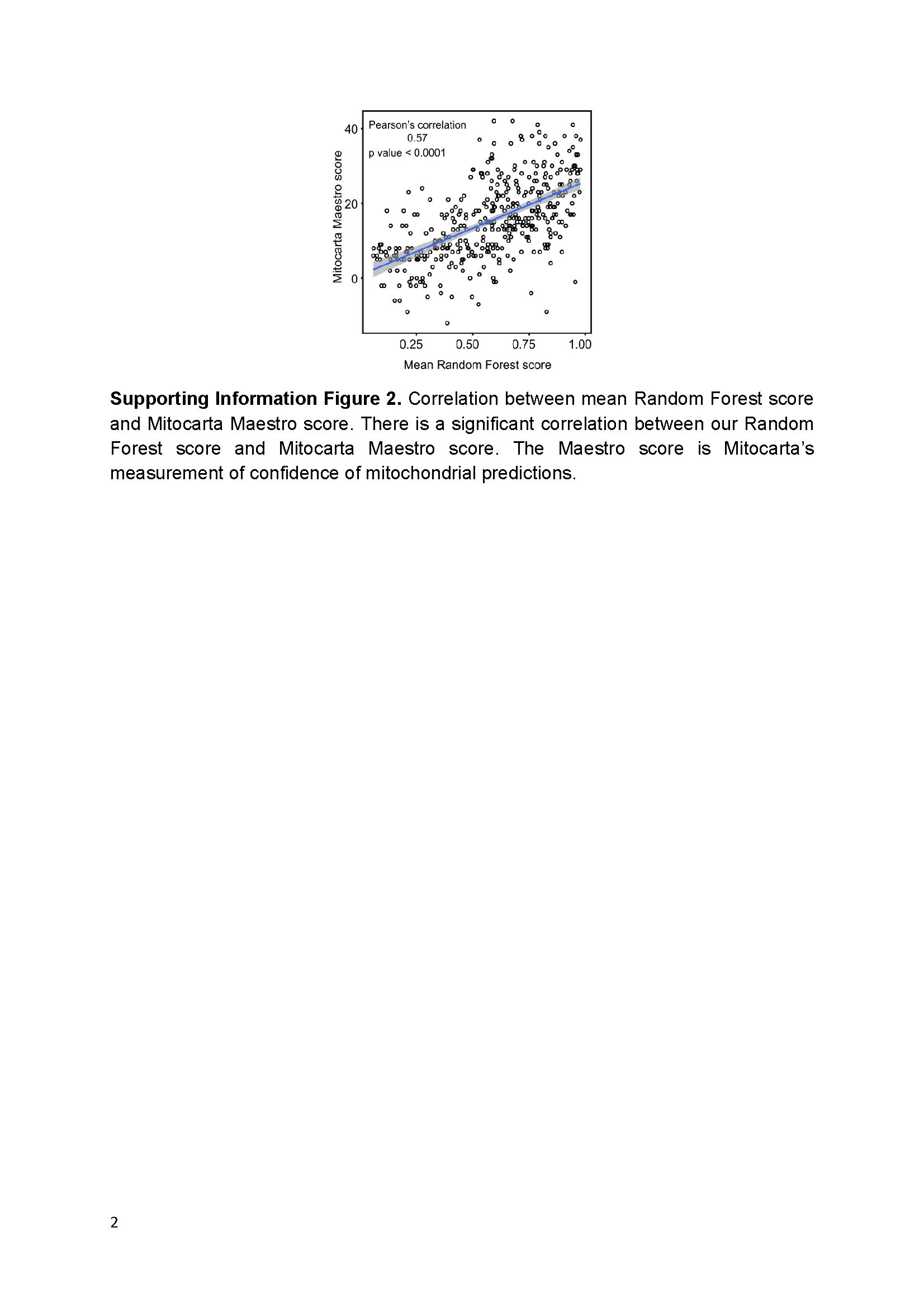

Supplement: Supplementary file 1 — Supplementary Material [file PMIC-16-393-s001.zip › pmic12175-sup-0002-Supporting_Informati.jpg]
